# The Pre-Polarization and Concentration of Cells near Micro-Electrodes Using AC Electric Fields Enhances the Electrical Cell Lysis in a Sessile Drop

**DOI:** 10.3390/bios15010022

**Published:** 2025-01-06

**Authors:** Kishor Kaphle, Dharmakeerthi Nawarathna

**Affiliations:** 1 Department of Electrical and Computer Engineering, North Dakota State University, Fargo, ND 58105, USA; kishor.kaphle@ndus.edu; 2 Biomedical Engineering Program, Department of Electrical and Computer Engineering, Old Dominion University, Norfolk, VA 23508, USA

**Keywords:** electric fields, cell lysis, induced transmembrane potential, dielectrophoresis and AC electroosmosis

## Abstract

Cell lysis is the starting step of many biomedical assays. Electric field-based cell lysis is widely used in many applications, including point-of-care (POC) applications, because it provides an easy one-step solution. Many electric field-based lysis methods utilize micro-electrodes to apply short electric pulses across cells. Unfortunately, these cell lysis devices produce relatively low cell lysis efficiency as electric fields do not reach a significant portion of cells in the sample. Additionally, the utility of syringe pumps for flow cells in and out of the microfluidics channel causes cell loss and low throughput cell lysis. To address these critical issues, we suspended the cells in a sessile drop and concentrated on the electrodes. We used low-frequency AC electric fields (1 Vpp, 0–100 kHz) to drive the cells effectively towards electrodes and lysed using a short pulse of 10 V. A post-lysis analysis was performed using a hemocytometer, UV-vis spectroscopy, and fluorescence imaging. The results show that the pre-electric polarization of cells, prior to applying short electrical pulses, enhances the cell lysis efficiency. Additionally, the application of AC electric fields to concentrate cells on the electrodes reduces the assay time to about 4 min. In this study, we demonstrated that low-frequency AC electric fields can be used to pre-polarize and concentrate cells near micro-electrodes and improve cell lysis efficiency. Due to the simplicity and rapid cell lysis, this method may be suitable for POC assay development.

## 1. Introduction

Global spending on healthcare has been constantly increasing and recently reached over USD 8.3 trillion. There is a huge demand for point-of-care (POC) systems, where screening, diagnostics, and treatments are carried out with a point of impact, e.g., in a mobile fashion, and provide rapid and low-cost healthcare solutions to reduce the current healthcare costs. A significant portion of medical screening and diagnostic methods involve examining the cell of interest. For example, the analysis of circulating tumor cells (e.g., invasively or non-invasively) is used in cancer detection and treatment monitoring. Cells are fundamental units that embody life-driving biomolecules and organelles enclosed in a semi-permeable thin phospholipid double layer collectively known as a cell membrane (4 to 8 nm thick) [1]. Cell membranes serve dual purposes: to barricade unwanted agents from entering the cell and to selectively enable the exchange of micronutrients and bio-products in the environment.

Achieving access to cells non-invasively helps the in vivo manipulation of cells, whereas cell-disruptive or invasive methods enable the harvesting of cell organelles and biomolecules of interest. One of the most common methods of non-invasively entering inside the cell is electroporation where the external electric field is applied in the cell sample to create pore(s) significant enough to inject a substance into the cells. This method is also known as reversible electroporation [1,2,3,4,5,6]. On the other hand, the cell-disruptive method of accessing the cell contents is commonly called cell lysis [4,7]. Cell lysis primarily relies on cell membrane breakdown either mechanically, chemically, or electrically [7,8].

Mechanical lysis is performed using shear force generators such as a high-pressure homogenizer and bead mill to rupture the cell membrane forcefully [9,10]. Although this method is highly efficient and not a cell-dependent technique, it suffers from serious functional disadvantages, such as excessive heat production that can potentially damage the cellular organelles, enzymes, and biomolecules of interest (e.g., mRNA and protein) [9,10,11]. Additionally, mechanical lysis is an expensive method requiring heavy equipment usage and tedious subsequent purification processes [8]. Chemical lysis is performed by using a dedicated lysis buffer such as alkali or detergent to disintegrate the cell membrane either by changing the pH of the membrane or by destabilizing the hydrophobic–hydrophilic interactions of the cell membrane molecules [12,13]. Unlike mechanical methods, this method enables the extraction of cell organelles and biomolecules of interest, such as proteins, DNA, RNAs, and enzymes, viably with low-to-no excessive heating. Nonetheless, it is a slow process; for instance, alkaline lysis may take up to 6 to 10 h to complete the lysis. Additionally, chemical lysis requires special reagents and a complicated post-lysis chemical cleaning process, making it sophisticated and expensive, especially for desirable settings such as POC systems [8,14].

The electrical lysis method involves applying brief electrical field pulses of AC or DC electric fields to produce cell membrane pore(s) bigger than the repairable pore size (~40 nm) of the cell, thereby leveraging osmotic pressure differences between cells and suspending media to burst the cell [1]. This method offers unique advantages over both the mechanical and chemical methods of lysis in terms of cost, flexibility, efficiency, and system integration for subsequent therapeutic processes [1,8,15,16,17]. The real perks of electrical lysis, however, are pronounced in its application for POC settings. There have been numerous developments in the adaptation of electrical lysis in POC devices for biomolecule (e.g., DNA and RNA) extraction and manipulation [1,3,6,18,19]. The majority of POC devices utilize micro or nano scale electrodes for cell lysis. The electric fields produced by micro or nanoelectrodes are generally in the short range and extend to a few hundred micro or nanometers from micro and nanoelectrodes, respectively. As a result, most cells in the sample are not exposed to electric fields and subsequently are not lysed. This technical deficiency needs to be addressed immediately as it produces very low cell lysis efficiency. Additionally, traditional POC cell lysis devices use microfluidics, where the cell sample is introduced to the lysis platform using automatic and semi-automatic infusion pumps [1,3,16]. While these microfluidic devices offer great advantages, such as flexible system integration and automation and the touch-free maneuvering of the target sample, cell lysis is a slow process due to its pumping rate constraints [1,16,20]. Hence, there is a need for a simpler, flexible, portable, and cheaper electrical lysis system that can be used in POC settings.

In this study, we focused on the cells suspended in a sessile drop to eliminate the need for infusion pumps. Additionally, to address the low cell lysis efficiency, we studied the effects of cell concentration near the electrodes prior to cell lysis. We employed the AC electric fields to drive the cell toward the electrodes. The AC electric fields produce dielectrophoretic effects (on cells) and AC electroosmosis (on the buffer). Therefore, AC electric fields produce a dielectrophoretic (DEP) force on the cells and AC electroosmotic fluid flow (or viscous drag force on the cells). The DEP force has emerged with enormous potential in biotechnology and therapeutics because of its high selectivity, touch-free maneuvering, and electrical characterization of samples for various applications [21]. Most common practices are found to use DEP for the proper positioning of target cells into the most effective electric field region [17,22]. However, there are limited studies on the efficient coupling of DEP and electrical lysis to achieve higher lysis efficiency [17,22,23], as the ramification of the full potential of the cascaded system like this remained significantly untapped and the efficacy of electrical lysis remained lower for non-microfluidic POC settings [1,6]. AC electroosmosis is also a well-developed concept in the literature. Many studies have focused on understanding the fluid flow produced by AC electroosmosis. This study investigates the utility of AC electric field effects on suspended cells and fluids for biomedical assay development (e.g., cell lysis).

## 2. Experimental Section

### 2.1. Electric Field and Electric Field Gradient Calculations

The AC/DC module of COMSOL Multiphysics (COMSOL Inc., Burlington MA, USA) was utilized to calculate electric fields and their gradients (∇*E*^2^). In these calculations, it was assumed that the cell sample was suspended in a buffer with 5.5 µS/cm, and a voltage of 1 Vpp was applied across each electrode in the interdigitated electrode pair. Additional details about these calculations are reported elsewhere [24].

### 2.2. Cell Sample Preparation

Commercially available single-donor human whole blood (Innovative Research Inc., Peary Court, MI, USA) was purchased and used in the experiments. First, red blood cells (RBC) were lysed using a commercially available RBC lysis buffer (Thermo Fisher Scientific, Waltham, MA, USA). Briefly, 1 mL of human blood was mixed with 10 mL of RBC lysis buffer and incubated at room temperature for 13 min before being centrifuged at 500× *g* for 5 min at room temperature. Next, the supernatant was removed, and the cell sample was resuspended in 500 µL of the 0.05 times diluted Tris EDTA (TE; conductivity ~6 µS/cm) buffer. Finally, 3 µL of the cell sample was pipetted on the electrode for lysis experiments. Live and dead cells were identified using Trypan blue staining. The manufacturer-provided protocol was used for staining.

### 2.3. Cell Concentration, Electric Field-Based Cell Lysis and Analyses

Commercially available interdigitated electrode devices (electrode spacing = 10 µm, width of the electrodes = 10 µm, and number of electrode pairs = 250; G-IDEAU10, Metrohm AG, Riverview, FL, USA) were used in the experiments. To electrically polarize and concentrate the cells toward electrodes, 1 Vpp 10 kHz or 1 Vpp 100 kHz was applied for 4 min using a commercially functioning generator (Tektronix, Beaverton, OR, USA). To lyse the concentrated cells, a 10 Vpp (1MV/m) 4 Hz square wave (with a pulse width of 100 µs) was applied for 2 s. Post-lysis analysis was performed using hemocytometry, UV-spectroscopy, and fluorescence imaging. Cell lysis efficiency was calculated by counting the initial cells that were pipetted on the electrodes and the remaining cells after applying cell concentration and cell lysis conditions. A schematic diagram showing the experimental steps is provided in Supplementary Figure S1.

### 2.4. Genomic DNA Concentration

The experiments related to the DNA concentration on the T-electrodes were carried out by adding EVA-green dye (Ex: 499 nm, Em: 526 nm; Biotium, Inc., Fremont, CA, USA) to the suspension. The experiments were carried out in two steps: First, the cell sample was pipetted on the interdigitated electrodes, 10 kHz was applied to the AC electric field (discussed above) to the concentrate cells on the electrodes, and cells were lysed by applying an electric pulse (discussed above). Second and finally, the sample was extracted by carefully pipetting from the electrode and was then deposited on interdigitated T-electrodes, and 3 MHz (10 Vpp) was applied for about 10–15 min. The details about T-electrodes, including the dimensions and manufacturing, can be found elsewhere [24]. Finally, fluorescence imaging on the concentrated DNA sample on the T-electrodes was performed using a fluorescence microscope with appropriate filters.

## 3. Results and Discussion

**Figure 1:**
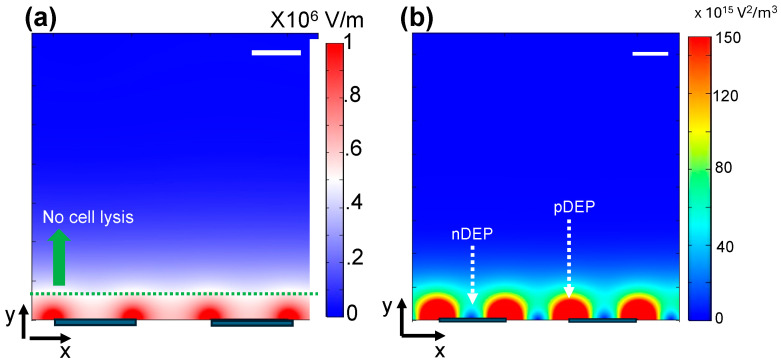
Calculated electric field (**a**) and electric field gradients (**b**) in the vicinity of the interdigitated electrodes used for cell lysis experiments. Scale bars indicate 5 µm.

First, we studied the manipulation of cells using electric fields. The electric field polarized the cells, and polarized cells experienced a DEP force when there was an electric field gradient (∇*E*^2^). For example, for a spherical cell suspended in a media with absolute permittivity (ε_m_), upon the application of the variable electric field (E), the DEP force is given by Equation (1) [21].

(1) $$F_{DEP}=2\pi \varepsilon_{m}R^{3}CM(\nabla E^{2})$$

where CM is a factor known as the Clausius–Mossotti factor (typical range −0.5 to 1), and its value depends on the permittivity and conductivity of the suspended particle (cell) and suspending media; R is the radius of the cell; and ε_m_ is the permittivity of the cell at the applied frequency of the electric field. Based on the frequency of the applied electric field, the conductivity, and dielectric permittivity of the cell and its surrounding buffer, the DEP force can attract the cells towards the highest ∇*E*^2^, known as positive DEP (pDEP) or push towards a lower ∇*E*^2^, known as negative DEP (nDEP). In the experiments, we varied the frequency of the electric field (0–20 MHz) while keeping the voltage at 1 Vpp (electric field ~1MV/m) and observed the motion (using bright field microscopy) of the cells around the electrodes. We set the voltage to 1 Vpp to minimize the cell damage due to potential electroporation. We found that cells experience nDEP around 10 kHz and pDEP around 100 kHz. To verify these observations, we calculated the CM factors of the cells (without RBCs) for 10 kHz and 100 kHz, respectively. Briefly, we used the calculated CM variation with frequency values reported by Piacentin et al. for the buffer conductivity value of 55 mS/m [25]. Moreover, the crossover frequency value for platelets in this buffer was calculated to be 2 MHz. Since the crossover frequency was directly proportional to the medium conductivity, we calculated the crossover frequency of the cells suspended in the buffer that we used to be around 20 kHz and the CM factors to be –0.5 and 1 for 10 kHz and 100 kHz, respectively. Therefore, our experimental observations were consistent with our CM factor calculations. To further understand the cell concentration mechanism, we calculated the electric field and ∇*E*^2^ (see Figure 1). It can be understood from Figure 1 that the electric fields, ∇*E^2^*, and DEP forces are very strong when the cells are located near the electrodes. Therefore, the DEP force produces a short-range effect on cell concentration. To effectively concentrate all cells in the sample on the electrodes, there must be other forces (or long-range forces) that can focus the cells on the DEP force active region. Those forces are the weight of the cell, buoyancy force, and viscous drag force. The viscous drag force is dependent on the fluid velocity, which is produced by AC electroosmosis.

Many studies have investigated the fluid motion by AC electroosmosis, and it was reported that the magnitude of the velocity depended on the applied frequency in relation to the characteristic frequency or $\frac{\sigma }{\varepsilon }$ (~125 kHz) of the buffer, where σ and ε are the conductivity and dielectric permittivity of the buffer, respectively. If the applied frequency is lower than the characteristic frequency, a strong AC electroosmosis flow takes place in the sample. Typically, for a low conductivity buffer like that which we suspended the cells with, the characteristic frequency is around 125 kHz. If the applied frequency is around or above the characteristic frequency, no significant fluid flow takes place in the sessile drop. We expected a varying degree of fluid flow in the frequencies that we used in the experiments. For example, at 10 kHz, a strong viscous drag force was produced on the cells due to AC electroosmosis. In contrast, at around 100 kHz, a weak viscous drag force was produced on the cells. Additionally, sedimentation force (the weight of the cells–buoyancy force) can also play a role in cell concentration, especially when the viscous drag force is weak. The time taken to concentrate cells on the electrodes depends on the balance of these forces. The time can be increased or decreased by changing the magnitude of the applied electric field and its frequency. On average, it took about 4 min to concentrate about 90% of the cells on the electrodes. Additionally, in evaporating droplets or sissle drops, capillary flow-based particle flow toward the edge of the droplet was possible. Moreover, capillary flow produced coffee ring formation, especially when molecules were suspended in the droplet. We did not experimentally observe the concentration of cells through the coffee ring effect.

Once the cells were concentrated on the electrodes, the AC electric field was turned off, and a short DC pulse was applied to destroy the cell membrane by destructive or irreversible electroporation. The external electric fields modified the intrinsic membrane potential of the cell. The contribution from the external field to membrane potential is called induced transmembrane potential (v_induced_). Mathematically, for a spherical cell, v_induced_ = f_e_ERcosΦ, where R is the cell radius, Φ is the angle between the pore and direction of the electric field, and f_e_ is the electroporation factor [26], which is given by the following:

(2) $$f_{e}=\frac{\sigma_{s}\left[3d_{m}R^{2}\sigma_{c}+\left(3{d_{m}}^{2}R-{d_{m}}^{3}\right)\left(\sigma_{m}-\sigma_{s}\right)\right]}{R^{3}\left(\sigma_{m}+2\sigma_{s}\right)\left(\sigma_{m}+{0.5\sigma }_{c}\right)-{\left(R-d_{m}\right)}^{3}(\sigma_{s}-\sigma_{m})(\sigma_{c}-\sigma_{m})}$$

where membrane thickness is d_m_, the electrical conductivity of cell contents is σ_c_, the conductivity of the cell membrane is σ_m_, and suspending media conductivity is σ_s_.

Previous studies have extensively used short DC pulses (e.g., µs to ms long) for cell lysis experiments. In addition, recently, AC electric fields have also been used for reversible electroporation. For example, recently, when primary T-cells were electroporated using AC electric fields, over 75% cell viability was achieved [27]. If the induced transmembrane electric potential is >1 mV, reversible electroporation destroys the cell membrane and lyses the cell. For the experimental conditions that we used (maximum electric potential of 10 Vpp or 1MV/m) in our experiments, we calculated the induced transmembrane potential values to be about 7.5 mV (maximum). This clearly shows that the electric fields used in the experiments can produce irreversible electroporation. The electric field distribution around the sample drop is location-dependent. We calculated the region near the electrode that could produce the irreversible electroporation (induce transmembrane potential >1mV) of the residing cells (green broken line in Figure 1). Note that cells must be located within about 5–7 µm from the electrodes. This calculation highlights the importance of cell concentration to improve cell lysis efficiency. Without cell concentration, the cells are located outside the lysis region and are not lysed.

**Figure 2:**
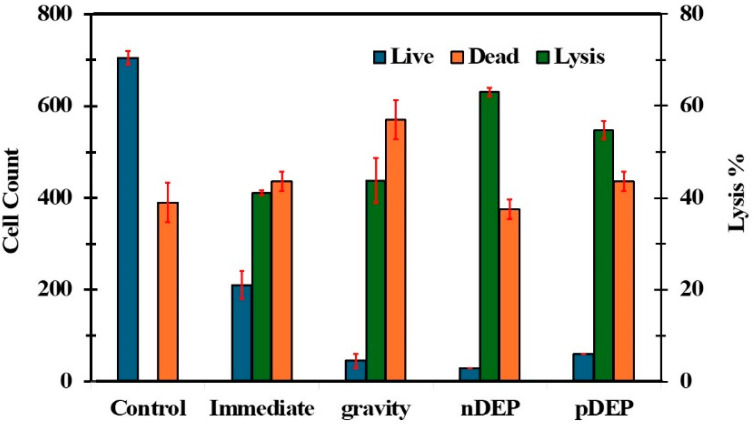
Variation in cell lysis efficiency and cell count (after application lysis electric field) with experimental conditions used in the study. Control: cells from the tube left at room temperature; Immediate: immediately after pipetting cells on the electrodes; Gravity: settling cells under gravity; nDEP: after applying negative DEP and pDEP: after applying positive DEP.

Throughout the literature, there are mainly three types of post-lysis analysis, namely cell counting (e.g., hemocytometry) [16,22], spectroscopy (e.g., UV-vis spectroscopy) [2,18], and imaging (e.g., fluorescence imaging) [6,16,28]. We used all three methods to evaluate the performance of the cell lysis experiments. First, we evaluated the lysis efficiency, live cell, and dead cell percentages after exposure to electric fields (Figure 2). Our results show that superior lysis efficiency was achieved when AC electric fields were used to concentrate the cells on the electrode. The highest lysis efficiency (~65%) was achieved when the 10 kHz AC electric fields were used to concentrate the cells. In contrast, when 100 kHz AC electric fields were used to concentrate the cell samples, ~55% lysis efficiency was achieved. By comparison, a larger CM factor (−0.5 vs. 0.1) was produced when 10 kHz or nDEP was used. The CM factor is directly related to the polarizability of the cell. Therefore, when 10 kHz was used, cells were more polarized than 100 kHz. The polarization in the low frequency is limited to the cell membrane, and the polarization increases the induced transmembrane potential. Our experimental results show that the pre-polarization of the cell membrane enhances cell lysis.

Additionally, when a 10 kHz frequency was applied, the speed of the buffer flow within the droplet was larger than that of 100 kHz. The buffer flow can produce better mixing at 10 kHz. Figure 1b shows the location of the cell concentration. ∇*E^2^* values of the nDEP and pDEP regions were 150 × 10^15^ and 20 × 10^15^ V^2^/m^3^, respectively. We roughly calculated the DEP forces produced on the cells by nDEP and pDEP to be almost equal. Therefore, superior cell lysis recorded for nDEP could be due to the pre-polarization produced by 10 kHz electric fields. The lowest number of dead cells (post-lysis) was recorded when AC electric fields (10 kHz or 100 kHz) were used to concentrate cells on the electrodes (see Figure 2). The dead cells could have been produced when cells were exposed insufficiently to the electric field strengths (inducing less than 1mV of induced transmembrane electric potential). For example, when a cell was located far away from the electrodes or outside the lysis region (Figure 1a), the induced transmembrane potentials were unable to lyse the cells, but it could kill the cells by electroporation. As noted from the data, when the cell sedimentation force (“gravity” bar in Figure 2) was used, poor cell lysis efficiency was produced because most cells were not located in the high electric field regions or outside the cell lysis region (Figure 1a).

**Figure 3:**
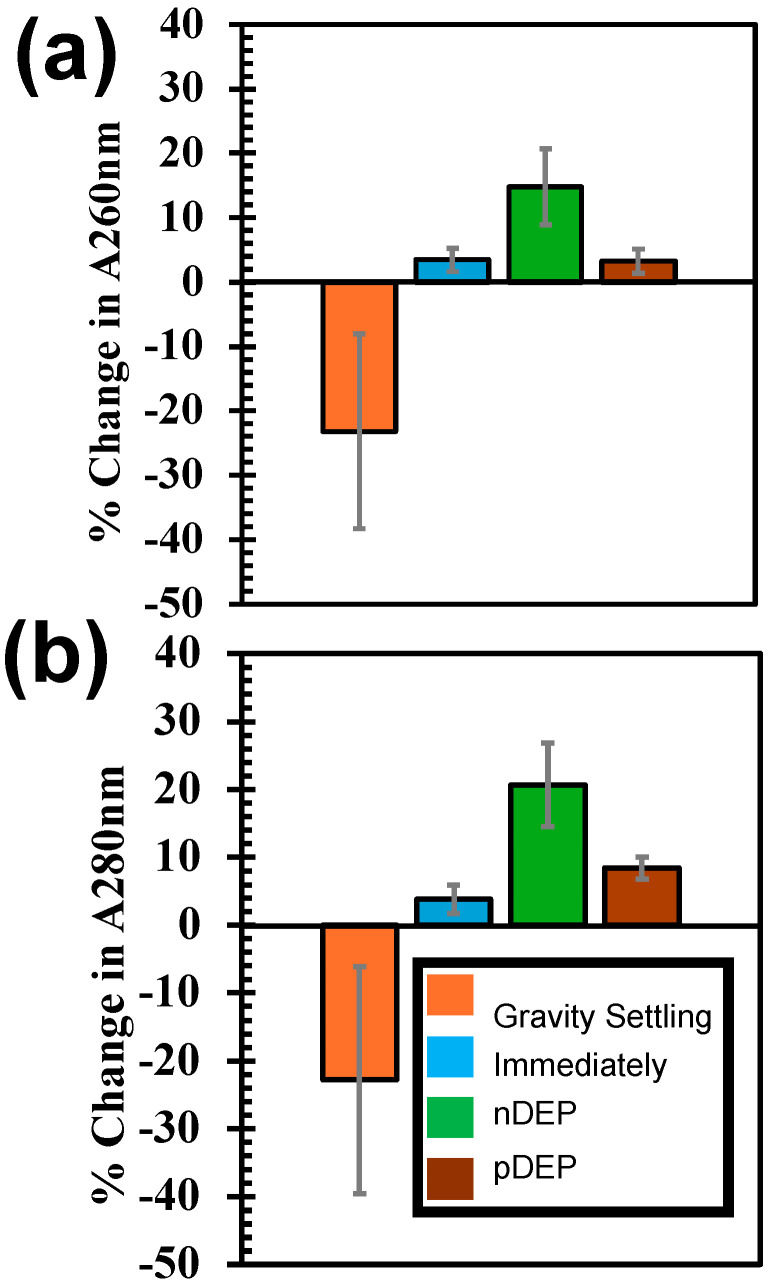
The quantification of nucleic acid (**a**) and protein molecules (**b**) in the buffer after concentrating cells on the electrodes using gravity settling, immediately after pipetting cell sample on the electrodes, applying nDEP, pDEP. For all these conditions, the cell samples were lysed applying 10 V pulse for 2 s.

Ideally, when cells are lysed, cellular contents flow into the buffer. We analyzed the molecules in the buffer pre- and post-cell lysis. We used UV-vis spectroscopy to quantify proteins and nucleic acid molecules. The absorbance values at 260 nm and 280 nm were used to quantify nucleic acid and protein molecules in the samples, respectively (Figure 3). A large change in the absorbance values was reported when the DEP forces were used to concentrate cells. Additionally, the greatest change in absorbance values was reported when 10 kHz was used to concentrate cells (Figure 3). As discussed previously, when 10 kHz was used, the highest lysis efficiency was reported. The higher the cell lysis efficiency, the higher the amount of protein and nucleic acid molecules it was expected to have, as observed in the experiments. Another issue with the application of electric fields is the degradation of the molecules, especially protein, RNA, and DNA molecules. If there is a significant degradation of molecules, we would expect either small or no change to the pre vs. post-lysis. A negative increase in molecules was reported when the sedimentation-based cell lysis was utilized (“gravity” bar in Figure 3). Sedimentation by cellular concentration is a slow process, and it randomly places cells. Additionally, sedimentation-based cell concentration takes time to reach the electrodes. If the electrical lysis condition is applied before cells reach the electrodes, those cells cannot be lysed.

Next, we used fluorescence imaging to visualize the genomic DNA molecules dissipated from lysed cells. Briefly, in the experiments, we used a 10 kHz AC electric field to concentrate cells on the electrodes and lysed cells using an electric short pulse, as outlined in the Materials and Method section. Once the cell lysis was complete, the sample was extracted from the electrodes and pipetted on the T-electrodes. To be able to visualize the DNA molecules, we labeled the DNA molecules with commercially available DNA-specific binding dye molecules. Moreover, we added dye molecules to the cell sample prior to the cell concentration and lysis. Additionally, we used the DEP force of DNA molecules (10 Vpp and 1 MHz) to selectively concentrate DNA molecules in T-electrodes. The T-electrodes are an array of interdigitated electrodes with T-shaped electrodes that were developed by our group for molecular concentration experiments. In previous studies, we demonstrated that T-electrodes are suitable for molecular concentration experiments using the DEP force because these electrodes produce the strong ∇*E*^2^ needed to efficiently and effectively concentrate molecules on the electrodes. Previously, we concentrated short RNA molecules (~22 bases long; 10 Vpp and 1–3 MHz) and antigen molecules (10 Vpp and 600 kHz) on T-electrodes using their DEP forces [29]. The ability to concentrate the genomic DNA molecules released from lysed cells using DNA-specific frequency provides additional proof.

**Figure 4:**
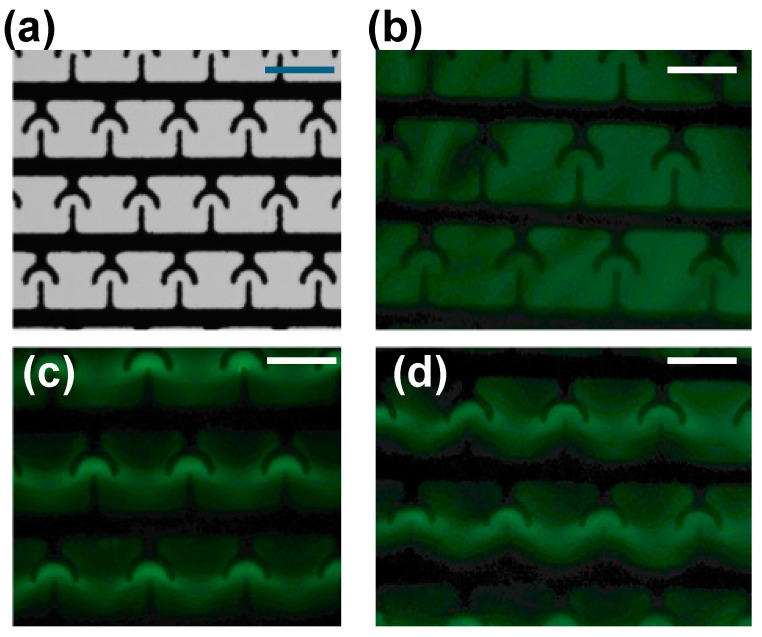
Concentration of cellular DNA on T-electrodes using AC electric fields. (**a**) Picture of interdigitated T-electrodes. (**b**–**d**) fluorescence images of T-electrodes after applying no electric potential, 10 Vpp (1 MHz), 10 Vpp (500 kHz), respectively. Scale bars show 10 µm.

**Figure 5:**
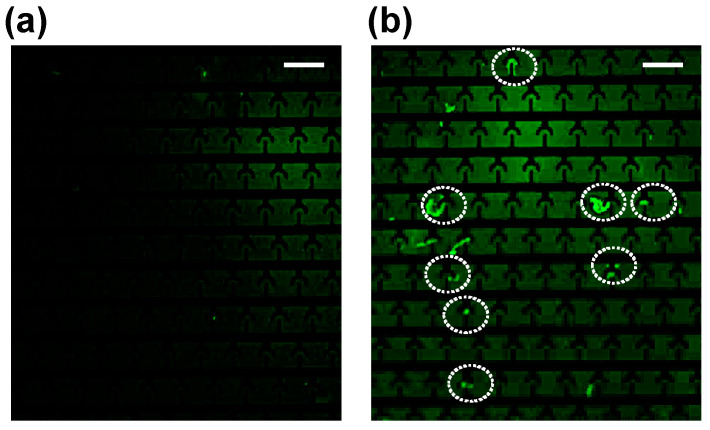
Concentration of cellular DNA on T-electrodes using AC electric fields (10 Vpp, 1 MHz). (**a**) Image of T-electrodes (from concentrated cells) without cell lysis. (**b**) Image of T-electrodes (from concentrated cells) on T-electrodes after electrical cell lysis. White circles show the concentrated DNA molecules on the electrodes. Scale bars show 20 µm.

First, to find the frequency needed for the cellular DNA concentration, we separately conducted a series of experiments using commercially available lambda DNA (λ-DNA; New England Biolabs, Ipswich, MA, USA) and varied the frequency of the electric field (0–20 MHz). Figure 4 shows the concentration of DNA on T-electrodes. λ-DNA was selected for the experiments because its length and linear nature resemble genomic DNA. Briefly, for each frequency value, we recorded an image, and the total fluorescence intensity of the image was calculated using custom code written in MATLAB (version: 9.12, Mathworks, Natick, MA, USA). We then determined the frequency (1 MHz) that produced the largest intensity value (or strongest DEP force). Since DNA molecules are concentrated in the areas of highest ∇*E*^2^, DNA molecules experience pDEP. We then used 1 MHz to concentrate the DNA molecules from the cell-lysed sample (Figure 5). The images (Figure 5) show before and after cell lysis conditions were applied. Bright fluorescence spots on T-electrodes (white circles drawn with broken lines; Figure 5b) were detected when DNA was released from lysed cells concentrated using 10 Vpp (1 MHz). Our experiential results illustrated in Figure 5 show that fluorescence accumulates in the high ∇*E*^2^ regions near the T-electrodes (see enclosed regions with broken white lines), which means that DNA molecules (labeled with green fluorescence dye) experienced pDEP and were attracted to these regions. This experimental data show that the DEP force produced using 10 Vpp (1 MHz) can be used to concentrate the DNA molecules on T-electrodes from the buffer. Furthermore, if needed, isolated DNA can be extracted from individual electrodes using a micropipette for further analysis or any other downstream applications.

## 4. Conclusions

In this study, we have demonstrated the utility of AC electric fields to electrically polarize and concentrate cells (suspended in a sessile drop) on electrodes. The results show that the pre-concentration of cells on electrodes enhances cell lysis. Additionally, this study demonstrated that pre-polarization by AC electric fields enhances lysis efficiency. No infusion pumps were used in the study for flow cell samples with the drop. Our data show that the buffer flow produced by the AC electroosmotic was sufficient to manipulate cells in the sessile drop. Alternatively, other fluid flow mechanisms (e.g., electrothermal flow) can also be used for the flow buffer within the sissle drop. We did not investigate the electrothermal flow as it requires a high conductivity buffer. The cell lysis assay time was about 4 min. This means that this assay can be used in POC settings for rapid DNA extraction. The DNA extraction at POC can be useful in many applications, identifying unknown diseases and rapidly spreading infections. Furthermore, a short isolation time is also advantageous as it can be extended to develop additional biomedical assays. For example, the DEP force can be used to selectively concentrate cells (e.g., tumor cells from a biopsy sample) of interest on the electrodes, lyse, and isolate DNA for further analysis. Our future studies will focus on utilizing cell lysis in more focused biomedical engineering and POC applications.

## Data Availability

The original contributions presented in this study are included in the article/Supplementary Material. Further inquiries can be directed to the corresponding author.

## Supplementary Materials

The following supporting information can be downloaded at: https://www.mdpi.com/article/10.3390/bios15010022/s1, Figure S1: Experimental steps used in the study. (a) First cells were pipetted on the electrode and formed a sissle drop. (b) Cells were pre-concentrated and pre-polarized. (c) Cells were lysed using electric fields.
