# Supplementary material for: The Pre-Polarization and Concentration of Cells near Micro-Electrodes Using AC Electric Fields Enhances the Electrical Cell Lysis in a Sessile Drop
Source: Biosensors (Basel). 2025 Jan 6;15(1):22. doi: 10.3390/bios15010022 (PMC11763957; doi:10.3390/bios15010022)
Supplement: Supplementary file 1 [file biosensors-15-00022-s001.zip › biosensors-3355726-supplementary.pdf]

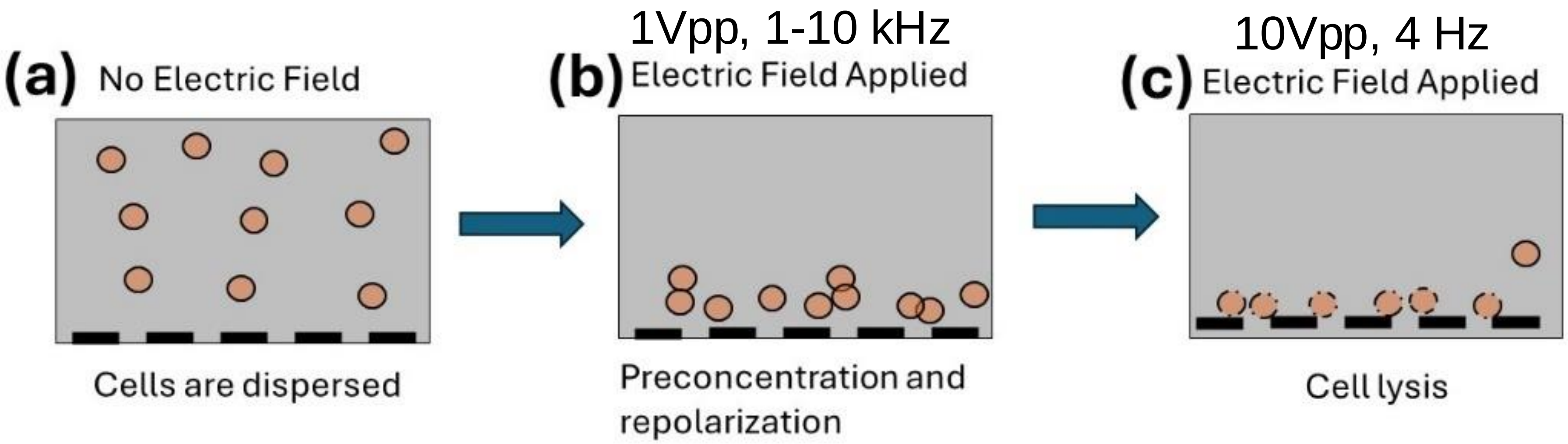

Supplementary figure S1

Experimental steps used in the study. **(a)** First cells were pipetted on the electrode and formed a sissle drop. **(b)** Cells were pre-concentrated and pre-polarized. **(c)** Cells were lysed using electric fields.
